# Quality of Life of Patients With Major Amputations in the Tertiary Care Center of Western Rajasthan: A Prospective Observational Study in 2019-2020

**DOI:** 10.7759/cureus.20419

**Published:** 2021-12-14

**Authors:** Ashok K Puranik, Souvik Maity, Satya P Meena, Archismita Santra, Mahendra Lodha, Mayank Badkur

**Affiliations:** 1 General Surgery, All India Institute of Medical Sciences, Jodhpur, Jodhpur, IND; 2 Centre for Community Medicine, All India Institute of Medical Sciences, New Delhi, Delhi, IND

**Keywords:** whoqol- bref, sf-12, quality of life, hospital stay, complications, amputations

## Abstract

Introduction

Amputation of a limb is a loss of physical integrity that has disastrous consequences for a person's mental, physical, and social well-being.

Aim

We aim to analyze the quality of life (QoL) after major amputations and long-term outcomes.

Method and materials

A prospective, observational study has been conducted in a health care institute in western Rajasthan from January 2019 to July 2020. This study included 64 patients who had major upper or lower limb amputations. We analyzed the sociodemographic factors of the patients, the type of procedure, postoperative hospital stay, complications, and follow-up status with both the SF-12 and the World Health Organization Quality of Life (WHOQOL)-BREF questionnaires. Mean, median, range, standard deviation, percentages, univariable, and multivariable logistic regression were analyzed with SPSS version 23.0 software (IBM Corp., Armonk, NY).

Results

The mean age of the study patients was 53.6 years (SD 2.6) and they were mostly male (71.9%). Atherosclerotic peripheral vascular disease (PVD) was the most common indication (37.5%) of amputation, and below-the-knee amputation (46.88%) was the most commonly performed procedure. There was a significant increment in both PCS (p-value= 0.001), MCS scores (p-value=0.0001) of SF-12 and physical (p-value=0.0001) and psychological domains (p-value=0.001) of the WHOQOL-BREF questionnaire in the postoperative period. A total of 83.9% of patients have used prostheses, and 15.6% had mortality.

Conclusions

Major amputations can significantly affect the quality of life of patients, and all efforts should be made to avoid factors that adversely affect their quality of life.

## Introduction

Major amputations are ­­­­­­­­­defined as amputations above the level of the ankle joint for the lower limb or wrist joint for the upper limb. Amputation, although it is sometimes performed as a life-saving measure, contributes to the loss of physical integrity. It may cause severe mental trauma, followed by increased morbidity and mortality, especially in the young population.

Two terms, health status (HS) and quality of life (QoL) are closely related. However, little literature suggests discrepancies between both HS and QOL. HS is an objective parameter, whereas QOL refers to the patient-centric subjective evaluation of one’s wellbeing [1]. The aim of this study was to assess the quality of life after major amputations and long-term outcomes.

## Materials and methods

Study design, duration, and setting

A prospective observational cohort study has been conducted between the periods of July 2019 and June 2020 in the Department of General Surgery at the All India Institute of Medical Sciences, Jodhpur, Rajasthan, India.

Study size and selection of subjects

For sample size calculation, we have used the World Health Organization Quality of Life (WHOQOL)-BREF scores at six months of follow-up from our reference study by Peters et al. [1]. Considering the comparison between two mean WHOQOL-BREF scores at baseline and six months of follow-up, the following formula has been used and the assumption of a 10% non-response rate and 10% mortality at the end of the follow-up period has been made:

n = (Zα/2 + Z β) 2 * 2 * σ2/d2

The sample size calculation using both WHOQOL-BREF physical scores and psychological scores comes out to be the highest at 59, after considering deaths and non-responders. Hence, approximately the target sample size was 64.

The study included 64 adult patients (aged more than 18 years) who underwent major limb amputation following peripheral vascular disease (PVD), diabetes mellitus (DM), trauma, and other infective causes. Patients who had hemiparesis or paraparesis following a cerebrovascular accident or traumatic spinal injury and malignancy cases were excluded from the study.

Objectives

The primary objective was to measure the quality of life and health status using the WHOQOL-BREF and SF-12 questionnaires, respectively. Secondary objectives were to determine all factors affecting the changes in quality of life, like an indication of amputation, postoperative complications or morbidity, hospital stay, and the frequency of prosthesis use among the amputees.

Study tools and techniques

A predesigned proforma was used to collect the demographic characteristics, indications for amputation, presence of comorbid conditions, and baseline investigations. Preoperatively, a baseline HS was measured using the SF-12 questionnaire. Postoperatively, patients were assessed for QOL and HS using the WHOQOL-BREF and SF-12 questionnaires at the second and sixth months. The Indian adapted version of the WHOQOL-BREF was used. An interviewer-structured WHOQOL-BREF questionnaire was used when it could not be self-administered [2]. At the six-monthly follow-up, it was determined whether the patient was using a prosthesis or not.

The SF-12 is a shortened version of the SF-36, which itself evolved from the Medical Outcomes Study (MOS). The SF-12 was created to reduce the burden of responsibility. It comprises 12 questions that cover eight domains of health, like limitations in physical activities because of health problems, social activities because of physical or emotional problems, usual role activities because of physical health or emotional problems and associated bodily pain, general mental health (psychological distress and well-being), general health perceptions, and vitality (energy and fatigue) [3]. A 12-question survey form was filled out by all participants and accordingly scored by a clinical researcher.

Validity and Reliability

When compared to the SF-36 in various patient groups varying in age, physical and mental health, the SF-12 scores were similar to the SF-36 but almost always had bigger standard errors. Another study compared the SF-12 to the SF-36 in treatments for congestive heart failure, sleep apnoea, and inguinal hernia. The authors found that the SF-12 agreed with the MCS and PCS of the SF-36, noting that the scores recorded the same level of health and change over time [4]. Sinha et al. conducted a study to establish the validity of the SF-36 questionnaire in the Indian general population. They concluded that the translated version of SF-36 is valid and reliable for use among the Indian population [5].

**Figure 1:**
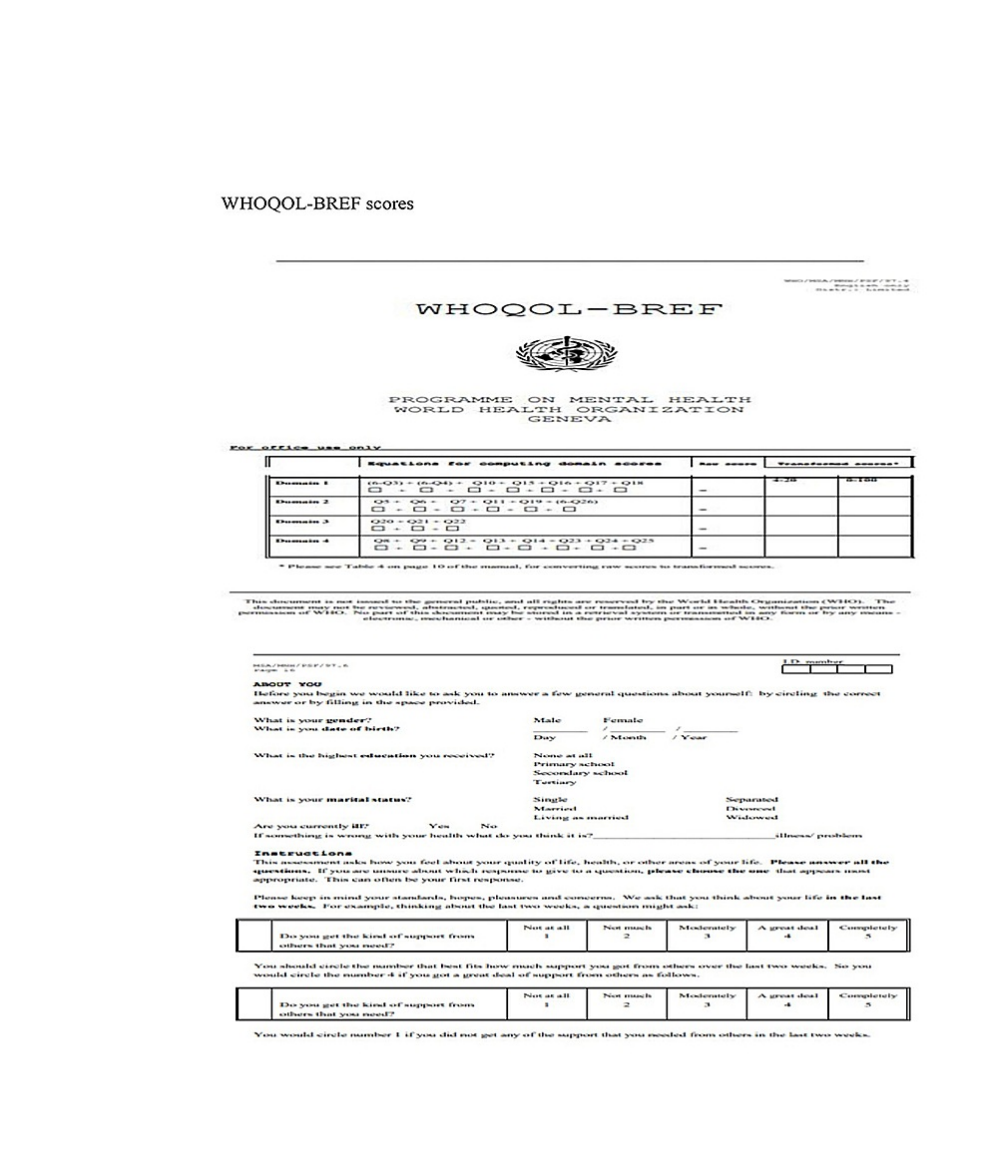
WHOQOL-BREF questionnaire

WHOQOL-BREF scores measure QOL in terms of four domains: physical, psychological, social, and environmental aspects. The physical health domain contains functional capacity, daily activities, items on mobility, energy, pain, and sleep cycle. The psychological domain measures include self-image, positive attitudes, negative thoughts, self-esteem, mentality, mental status, learning ability, religion, and memory concentration. The social relationships domain includes questions on personal relationships, sexual life, and social support. The environmental health domain measures problems related to safety, health, social services, financial resources living physical environment, transportation, general environment (noise, air pollution, etc.), recreation, opportunities to acquire new skills, and knowledge (Figure 1).

WHO's initiative to develop a quality of life assessment arises from a need for a genuinely international measure of the quality of life and a commitment to the continued promotion of a holistic approach to health and health care [6].

Statistical analysis

The data were tabulated in an excel spreadsheet before being analyzed with the IBM Statistical Package for the Social Sciences (SPSS) for Windows, version 23.0 (IBM Corp, Armonk, New York, US). Continuous variables and categorical data were described using mean, median, range, standard deviation, frequency, and percentages. Comparison between the SF-12 scores of the preoperative, second, and sixth months was done using the paired t-test. Univariable and multivariable logistic regression were used to show the association of all four factors, social, environmental, physical, and psychological domains, with WHOQOL-BREF scores. The association between the postoperative duration of stay and the SF-12 score in the second month was shown by linear regression.

Ethical considerations

The study was started after getting ethical clearance from the institutional ethical committee (AIIMS/IEC/2018/787). Informed consent was obtained before the data collection.

## Results

A total of 64 patients underwent major limb amputations within the study period. The mean age among the participants was 53.6 (SD±2.6) years, with a higher percentage of patients who were operated on in the elderly age group (>60 years). Diabetes was the most commonly observed comorbidity in study participants, especially in critical limb ischemia (CLI) patients, among whom 60% were diabetic. The most common indication for major limb amputation was limb ischemia or gangrene resulting from peripheral arterial disease (37.5%), followed by sepsis (21.9%) and crush injuries with or without vascular compromise (18.7%). In this study, 39.1% of participants had revision surgeries due to stump-related complications in the postoperative period, four patients had mortality in the same hospital stay, and eight patients expired in the follow-up period. All patients were encouraged to use prostheses, and of those, 83.9% of patients started using the prosthesis (Table 1).

**Table 1:** Demographic profile of patients

	Frequency (N=64)	Percentage (%)
Gender		
Male	46	71.9
Female	18	28.1
Age (in years)		
18–39	18	28.1
40–59	15	23.4
≥60	31	48.5
Married	60	93.7
Comorbidities		
None	29	45.2
Diabetes	15	23.3
Hypertension	4	6.2
Others (AKI, CAD)	4	6.2
More than one comorbidity	12	19.1
Addiction		
No	36	56.2
Yes	28	43.8
Indication for amputation		
Acute ischemia	6	9.4
Chronic ischemia	24	37.5
Trauma	12	18.7
Infections	14	21.9
Malignancy	8	12.5
Revision surgeries required		
No	39	60.9
Yes	25	39.1
Prosthesis given	N=56	
Yes	47	83.9
No	9	16.1

The mean postoperative duration was 9.6 days [(SD 0.8) 95% CI 8.0-11]. The mean PCS-12 and MCS-12 scores were calculated from the SF-12 questionnaire, and then the difference between the PCS-12 and MCS-12 scores between the second month and sixth month was calculated. There is no significant difference between preoperative PCS-12 scores and those at two months (mean difference −1.6, p-value =0.8). However, there is a significant increase in the physical component scores from the second month to the sixth month (mean difference 4.9, p-value<0.001). In the mental component score, no significant difference was found between the preoperative and the second-month scores (mean difference 0.4, p-value=0.4) but there was a significant increase in score from the second to the sixth month (mean difference 6.1, p-value<0.001). The difference between the preoperative and the sixth-month values were also significant (mean difference 5.3, p-value= 0.002; Table 2).

**Table 2:** Difference in SF-12 from preoperative period to six months post-surgery PCS: physical component summary score, MCS: mental component summary score

	Mean difference	95% CI	p-value
Difference in PCS-12			
Preoperative stage and second-month post-surgery (N=60)	−1.6	−4.9 to 1.6	0.8
The second-month and sixth-month post-surgery (N=56)	4.9	2.6–7.1	<0.001
Preoperative stage and sixth-month post-surgery (N=56)	2.4	−1.5 to 6.4	0.1
Difference in MCS-12			
Preoperative stage and second-month post-surgery (N=60)	0.3	−3.8 to 4.4	0.4
The second-month and sixth-month post-surgery (N=56)	6.1	3.1–9.0	<0.001
Preoperative stage and sixth-month post-surgery (N=56)	5.3	2.1–8.6	0.002

WHOQOL-BREF has four domains, namely physical, psychological, social, and environmental. The study has calculated the mean differences in the scores of the second and sixth months of every domain. This duration gap provides an idea about a short-term QoL after amputation and the QoL after acquiring a prosthesis at six months. There was a significant difference between the scores of the second and sixth months in the physical domain (mean difference 12.7, p-value<0.001). There was also a significant increase in the psychological domain score from the second to the sixth month (mean difference 8.7, p-value<0.001). In the social and environmental domains, there was no significant increase in scores from the second to sixth month (Table 3).

**Table 3:** Differences in the WHOQOL scores in second-month and sixth-month post-surgery

Domain (N=56)	Mean difference	95% CI	p-value
Physical	12.7	7.7–17.8	0.0001
Psychological	8.7	3.3–14.1	0.001
Social domain	2.6	−1.1 to 6.2	0.08
Environmental domain	1.2	−15.1 to 17.5	0.40

Those who had any type of addiction had significantly times lower odds (AOR =0.2, p-value=0.03) of having a good score (≥60) in the WHOQOL-BREF physical domain six months post-amputation. Those who had undergone traumatic amputation had significantly lower odds (AOR= 0.3, p-value=0.01) of having a good score (≥60) in the psychological domain of WHOQOL-BREF at six months post-amputation.

With a unit increase in post-operative duration of stay, PCS-12 at the second month significantly decreased by −0.8 (p-value= 0.007) and MCS-12 significantly decreased by −0.5 (p-value=0.03). However, with a unit increase in the postoperative duration of stay, the WHOQOL-BREF physical domain score and psychological domain at the second month decreased by −0.7 which was not significant (Table 4).

**Table 4:** Association of postoperative stay in hospital with various domains at second-month post-surgery PCS: physical component summary score, MCS: mental component summary score

Postoperative duration of stay	Beta coefficient	95% CI	p-value
PCS-12	−0.8	−1.5, −0.2	0.007
MCS-12	−0.5	−0.9, −0.03	0.03
WHOQOL-BREF physical domain score	−0.7	−1.4, 0.02	0.06
WHOQOL-BREF psychological domain score	−0.7	−1.7, 0.2	0.1

Of the total, 39 patients had no complications, 24 patients had wound infections, 3 had stump gangrene, and 2 patients had sepsis. Four patients had in-hospital mortality among the 64 patients (mortality rate: 6.25%). A total of 17 patients required readmissions for wound closure or stump revision. Preoperative employment status was 40 out of 64 patients (62.5%) were unemployed at the time of admission.

Fifty patients had completed one-year follow-up after surgery, and four patients had lost to follow-up. Four patients died within six months, and two died from six months to one year of follow-up. The mean age group of 16.1% of patients who were not using a prosthesis was 64.67. Twenty-four patients had returned to work; out of those two employed patients lost their jobs.

## Discussion

The quality of life of an individual, which is a subjective appraisal of well-being, becomes more important than an objective assessment of health. Many studies have been conducted worldwide, especially in the western world, to evaluate the QOL of amputees, especially those undergoing amputation for CLI [1]. There are unique challenges in the field of health care faced by developing nations due to a lack of knowledge regarding disease progression and the cost factor, which may often be unbearable for needy patients in rural areas [6].

A systematic review by Hawkins et al. has shown the validation of the SF-12 questionnaire for measuring the HS of amputee patients due to vascular causes [7]. However, the WHOQOL-BREF questionnaire provides a chance to assess a patient’s physical, psychological, and social functioning. In contrast to SF-12, the WHOQOL-BREF questionnaire evaluates the patient’s perception by asking whether the patient is satisfied with his or her ability to carry out a certain task.

The SF-12 questionnaire was adopted from the SF-36 questionnaire to reduce the burden of responsibility. It uses the same eight domains and can be applied to the same group of people. However, the SF-12 questionnaire was based on the health professional’s definitions of QoL. SF-12 hence measures health from an objective viewpoint, whereas QoL accesses if patients are subjectively restricted in daily life. Especially in patients who underwent amputation due to peripheral vascular disease, living without pain is a greater priority than mobility. This results in a different perspective of QoL, which is difficult to measure. Few studies have been conducted to determine the domains that influence the quality of life [4,5,8,9].

Active participation of patients in society is often restricted by limitations in body structure and post-amputation functional status. However, personal and environmental factors play significant roles in determining post-amputation functional outcomes as well as related complications [10]. Psychosocial support may be an important determinant for adjustment to amputation [11]. There will be no improvement in the scores in the social and environmental domains. Thus, it can be interpreted that there may be an improvement in the mobility, functional capacity, and physical energy of patients over time from the second to the sixth month. This can also be correlated with the SF-12 score, where we found a significant improvement in the physical component score from the second to the sixth month. We have found that there is also an improvement in the psychological score, which correlates with increased self-esteem and mental status. This finding correlates with the SF-12 score obtained by us, where there was a significant improvement in the MCS-12 score in the sixth month.

We have analyzed the independent variables which favor or hinder the possibility of having a good score in these two domains. The cut-off age for a good quality of life score was taken to be 60, which has previously been discussed in other studies [12].

The immediate reaction to the prospect of amputation may vary and depends upon the etiology and circumstances, whether due to chronic medical illness, acute infections, or trauma. The context of amputation affects the psychological sequelae during the rehabilitation phase as well [13]. Patient anxiety often increases with depression after getting information regarding the planned amputation. Since this anxiety may be generalized or result in disturbed sleep and irritability, preoperative and postoperative counseling are very important [14].

Post-traumatic stress disorder is more common among individuals suffering from accidental trauma [15]. Traumatic amputees who come across amputations in the wake of sudden trauma have little time to experience the various stages of grief [16]. Gozaydinoglu et al. have studied mutual associations between body image perception, compliance with the prosthesis, and cognitive performance in transfemoral amputees after major trauma. They have shown that increased satisfaction with physical appearance in amputees decreases the effects of the negative outcomes related to depression, stress, social isolation, sleep problems, and pain problems [17]. Thus, a poor score among traumatic amputees may be due to the result of a poor adaptation to self-image after trauma. In contrast, post-traumatic stress disorder (PTSD) is relatively rare (<5%) among amputees whose surgery follows a chronic illness [18].

The most common cause of amputation in this study was chronic limb ischemia as a result of peripheral arterial disease (37.5%). Grudziak et al. reported the percentage of gangrene-related amputations to be 45.6% [19]. Dedicated trauma centers are expected to report traumatic amputations as the most commonly performed procedure [20].

Below-knee amputations are the most commonly performed procedure worldwide. The rising consensus regarding limb preserving surgeries has helped in reducing the level of amputation in modern times [21-23].

Smoking

Smoking is a common independent risk factor among amputees, especially among patients with atherosclerotic disease. It was found that patients who had any kind of substance abuse had significantly lower odds of having a good score (>60) in the physical domain. According to the study by Cui et al., smoking is associated with a loss of health-related quality of life (HRQoL) and an economic burden [24].

Steunenberg et al. reported that various treatment modalities like endovascular procedures and surgical revascularization before planned amputation increased the HS and QoL [25]. Albers et al. compared amputation versus surgical revascularization reporting inferior results regarding the amputation group. HS after primary amputation was superior when compared with secondary amputation [26]. This study has shown that there was a significant improvement in the HS from the second to the sixth month, but not from the preoperative to the second month. Thus, we concluded that improvement in health status is achieved over the long term rather than the short term. This is in concordance with studies that have shown significantly improved HS after amputation, with exceptions in the social domain in the long term [27].

Improvement in quality of life in amputees may be affected by physician-controlled factors like timing or level of amputation, informed decision making, post-amputation rehabilitation support, and a few patient factors like age, accompanying chronic diseases, mobility impairment, and prosthetic compliance.

Most of the patients in this study (62.5%) were unemployed at the time of admission and had poor to average socio-economic conditions. The adult population (40-59 years old) had lost work due to the disease. Thus, amputation has a significant impact on employability, and this should be addressed by vocational rehabilitation and other means. However, the study by Labroca et al. on patients with symmetrical peripheral gangrene with major amputations of limbs reported that despite multiple amputations, no patients were bedridden in the long term [28].

The mean hospital stay and in-hospital mortality rate in this study were considerably lower. The longer postoperative stay was found to be detrimental to the short-term health status (after two months) in both the physical and mental domains. From this observation, we conclude that objective measures can differ in the short-term post-amputation; however, a different subjective appraisal is attained only over time. We have also shown that around 83.9% of participants received prostheses. Those who did not receive prostheses belonged to the elderly group. This might be due to societal neglect towards the elderly population, which must be addressed during the rehabilitation phase [29]. Our study did not reveal any significant correlation between prosthesis use and QoL. However, studies have reported that the use of assistive devices affects the physical component score more than the mental score in terms of better outcomes [30].

Wound infections may lead to prolonged hospital stays due to the requirement for multiple revision surgeries, thus negatively affecting the health status postoperatively. The presence of diabetes, low albumin levels, and higher HBA1c are associated with postoperative complications and lead to lower PCS and MCS scores [30].

This study found an improvement in both the HS and QoL in major amputees over time. Therefore, there has been an improvement in both the objective and subjective well-being of the patient. Health care providers may improve patient care by assessing all the factors associated with physical and psychosocial adjustment during the perioperative period of an amputation procedure or prosthetic intervention. This is the hypothesis that a few factors, like the encouragement of better self-care, lower dependence, more social interaction, less isolation, and the promotion of preventive actions, can improve the functional capacity and quality of life after amputation.

To date, Indian studies regarding the quality of life of amputees are lacking. This is expected to focus on potential areas for strengthening the healthcare system and also on the efficiency of currently practiced rehabilitative measures.

Limitations

Long-term compliance with prosthesis use and prosthesis-related problems have not been included in the study. QoL analysis between the patients who had early or late prostheses and those who had no prosthesis was not compared. This does not perform subgroup analysis on the mortality rate according to the etiology of amputation. Additionally, the economic burden due to major limb loss has not been studied. Also, due to the COVID-19 pandemic, a significant number of patients were lost to follow-up.

## Conclusions

Physical rehabilitation remains poor among smokers, alcoholics, opium-addicted patients, and traumatic amputees who face problems with psychological adjustments. Critical limb ischemia following PVD remains the most common etiology for major limb amputation. Diabetes and smoking are important risk factors for PVD. Stump infection remains the most common postoperative complication, especially in diabetic patients, which leads to a prolonged postoperative hospital stay and hinders the improvement in short-term health status. Major amputations can significantly affect the quality of life of patients, and all efforts should be made to avoid factors that adversely affect their quality of life.
